# Correction to “Maxillary impacted canine replacing a central incisor with root resorption: Multidisciplinary treatment in a preadolescent patient”

**DOI:** 10.1002/ccr3.9455

**Published:** 2024-09-20

Wen LM, Song YY, Liu JN, Zhu Y, Huang XF. Maxillary impacted canine replacing a central incisor with root resorption: Multidisciplinary treatment in a preadolescent patient. Clin Case Rep. 2024 Jul 31;12(8):e9230.

In the published article entitled, “Maxillary impacted canine replacing a central incisor with root resorption: Multidisciplinary treatment in a preadolescent patient”, there was an error in one of the affiliations. The text “1 Department of Stomatology Friendship Hospital, Capital Medical University, Beijing, China.” and “Correspondence Xiao‐Feng Huang, Department of Stomatology, Friendship Hospital, Capital Medical University, Beijing 100050, China. Email: huangxf1998@163.com should read “1 Department of Stomatology, **Beijing** Friendship Hospital, Capital Medical University, Beijing, China.” and “Correspondence Xiao‐Feng Huang, Department of Stomatology, **Beijing** Friendship Hospital, Capital Medical University, Beijing 100050, China. Email: huangxf1998@163.com

We apologize for this error.

